# Familial Screening for the Prevention of Rare Diseases: A Focus on Lipodystrophy in Southern Saudi Arabia

**DOI:** 10.1007/s44197-023-00182-5

**Published:** 2024-01-17

**Authors:** Adel Abuzenadah, Nofe Alganmi, Raghad AlQurashi, Esraa Hawsa, Abdullah AlOtibi, Abdulrahman Hummadi, Ahmed Ali Nahari, Somaya AlZelaye, Nasser R. Aljuhani, Manal Al-Attas, Heba Abusamra, Shereen Turkistany, Sajjad Karim, Zeenat Mirza, Mohammed Al-Qahtani, Adeel Chaudhary, Mariam M. Al Eissa

**Affiliations:** 1 Faculty of Applied Medical Sciences, Center of Excellence in Genomic Medicine Research, King Abdulaziz University, 21589 Jeddah, Saudi Arabia; 2 Computer Science Department, Faculty of Computing and Information Technology, King Abdulaziz University, 21589 Jeddah, Saudi Arabia; 3 Molecular Genetics Laboratory, Public Health Laboratory, Public Health Authority, Riyadh, Saudi Arabia; 4 Jazan Endocrinology and Diabetes Center, Ministry of Health, Jazan, Saudi Arabia; 5 Pediatric Department, King Fahd Hospital, Jazan, Saudi Arabia; 6 Centre of Endocrinology and Diabetes Mellitus, Al-Qunfudah General Hospital, Al-Qunfudah, Makkah Province Saudi Arabia; 7 Department of Medicine Endocrinology and Diabetes, East Jeddah Hospital, Jeddah, Saudi Arabia; 8 Department of Medical Lab Technology, Faculty of Applied Medical Sciences, King Abdulaziz University, 21589 Jeddah, Saudi Arabia; 9 King Fahd Medical Research Center, Faculty of Applied Medical Sciences, King Abdulaziz University, 21589 Jeddah, Saudi Arabia; 10 Center of Innovation in Personalized Medicine, King Abdulaziz University, 21589 Jeddah, Saudi Arabia; 11 Medical School, AlFaisal University, Riyadh, Saudi Arabia

**Keywords:** Lipodystrophy, Common variants, Rare diseases, Saudi Arabia, Prevention

## Abstract

**Background:**

Lipodystrophy is a relatively rare, complex disease characterised by a deficiency of adipose tissue and can present as either generalised lipodystrophy (GLD) or partial lipodystrophy (PLD). The prevalence of this disease varies by region. This study aimed to identify the genetic variations associated with lipodystrophy in the southern part of Saudi Arabia.

**Methodology:**

We conducted a retrospective study by recruiting nine patients from six families, recruiting the proband whole exome sequencing results or any other genetic test results, screening other family members using Sanger sequencing and analysing the carrier status of the latter. These patients were recruited from the Endocrinology and Diabetes Clinic at Jazan General Hospital and East Jeddah Hospital, both in the Kingdom of Saudi Arabia.

**Result:**

Eight patients were diagnosed with GLD, and one was diagnosed with PLD. Of the six families, four were consanguineously married from the same tribe, while the remaining belonged to the same clan. The majority of GLD patients had an AGPAT2 c.158del mutation, but some had a BSCL2 c.942dup mutation. The single PLD case had a PPARG c.1024C > T mutation but no family history of the disease. In all families evaluated in this study, some family members were confirmed to be carriers of the mutation observed in the corresponding patient.

**Conclusion:**

Familial screening of relatives of patients with rare, autosomal recessive diseases, such as lipodystrophy, especially when there is a family history, allows the implementation of measures to prevent the onset or reduced severity of disease and reduces the chances of the pathogenic allele being passed onto future generations. Creating a national registry of patients with genetic diseases and carriers of familial pathogenic alleles will allow the assessment of preventive measures and accelerate disease intervention via gene therapy.

**Supplementary Information:**

The online version contains supplementary material available at 10.1007/s44197-023-00182-5.

## Introduction

Lipodystrophies comprise a rare, complex group of diseases characterised by a deficiency of adipose tissue. These conditions are often misdiagnosed or delayed, largely because of the obscure nature of these disorders [2]. Different versions of lipodystrophy can be distinguished by the distribution of adipose tissue loss as either generalised, localised or partial [3, 15]. The disease can present as generalised lipodystrophy (GLD) or partial lipodystrophy (PLD), which are differentiated based on the extent and location of adipose tissue loss [3, 10, 19]. Both forms can be acquired or have a genetic cause in the case of congenital or familial versions [3, 19]. For example, in familial PLD type 2, subcutaneous fat is lost from the upper and lower limbs, trunk and gluteal regions, but it can accumulate in the face and neck (OMIM entry 151,660) [9, 16]. The global prevalences of GLD and PLD are 0.96 and 1.67 per million individuals, respectively [6]. The disorder is diagnosed partly based on the loss of subcutaneous adipose tissue without evidence of an underlying catabolic state or nutritional deprivation [5]. Loss of adipose tissue can lead to decreased leptin levels, which can interfere with hunger–satiety signals, resulting in hyperphagia [7, 9]. The excess calories consumed from overeating are abnormally and inappropriately stored in the liver and muscle, which causes insulin resistance, hepatic steatosis and hypertriglyceridemia [2, 7]. Therefore, lipodystrophy is associated with a severe form of metabolic syndrome, including not only severe hypertriglyceridemia but also dyslipidemia, extreme insulin resistance and low levels of high-density lipoprotein C [7]. Additionally, to use these symptoms (and loss of subcutaneous fat) to diagnose lipodystrophy, decreased leptin levels can be used as a differential diagnostic test, in which very low fasting serum adiponectin and leptin levels indicate a diagnosis of GLD instead of PLD and correlate with hypertriglyceridemia, high insulin levels (or insulin-resistant diabetes) and low high-density lipoprotein C [10]. Therefore, GLD and PLD can be considered distinctive types of lipodystrophy.

The various forms of lipodystrophy can be differentiated based on whether they are acquired or inherited. Moreover, there is phenotypic and genetic heterogeneity, even among the congenital or familial forms of lipodystrophy [19]. Different mutations in the same gene can give rise to different characteristic features, and mutations in multiple genes can cause congenital or familial lipodystrophy [3]. For example, peroxisome proliferator-activated receptor gamma (*PPARG*) is a nuclear receptor involved in insulin resistance, lipid metabolism and inflammation, and mutations in the corresponding gene are linked to PLD [21]. Mutations in the genes encoding lamin A (*LMNA*), cell death-inducing dffa-like effector C (*CIDEC*) and perilipin 1A (*PLIN1A*) are associated with PLD, whereas mutations in genes encoding 1-acylglycerol-3-phosphate-O-acyltransferase 2 (*AGPAT2*), caveolin 1 (*CAV1*) and polymerase-I-and-transcript release factor (*PTRF*), as well as in the Berardinelli–Seip congenital lipodystrophy 2 gene (*BSCL2*), have been found in patients with congenital GLD [15]. Overall, a variety of mutations in diverse genes are present in different populations, including rare or even novel mutations [12]. For example, in Saudi Arabia, mutations in *PTRF* have been discovered in patients with lipodystrophy, including the novel *PTRF* c.550G > T; p.Glu184*, a nonsense mutation that truncates the protein [12]. Another case report determined that the *AGPAT2* c.158del/p.Gly53Alafs*8 mutation caused lipodystrophy in two Saudi siblings [11].

This research focused on the genetics associated with lipodystrophy in the Saudi population and determined how implementing familial carriers screening for rare disease (RD) could reduce future incidences while furthering understanding of lipodystrophy’s genetic background and comparing it to mutations presented worldwide.

## Materials and Methods

### Ethical Approval

The Institutional Review Board of the Jazan Health Research Ethics Committee, Ministry of Health, Saudi Arabia, approved this study. Approval number IRB-No.2275 was received for one year starting September 2022. Project number IFPNC-008–141-2020 was funded by the Deputyship for Research and Innovation, Ministry of Education in Saudi Arabia and King Abdulaziz University, DSR, Jeddah, Saudi Arabia.

### Study Design

This study was conducted retrospectively on patients diagnosed with GLD or PLD. The data included genetics and clinical phenotypics, which were recruited from Jazan General Hospital. One patient and family members were from East Jeddah Hospital, Kingdom of Saudi Arabia. The inclusion criteria were all patients diagnosed with GLD or PLD, and the initial diagnosis was confirmed with molecular genetics using next-generation sequencing technology at a commercial laboratory accredited by the College of American Pathologists. We further screened other family members, recorded any phenotype and obtained consent for genetic testing using Sanger sequencing. We excluded patients with no initial genetic testing in their primary diagnosis of GLD or PLD.

### Data Collection

The data, including patient demographics, family history, and genetic test results, were collected from the diabetes clinic to confirm inheritance patterns. We took a 5 ml blood sample in an EDTA tube and sent it to the Centre of Excellence in Genomic Medical Research (CEGMR) at King Abdulaziz University. The received blood was processed, and the molecular derivatives were isolated and stored in the biobank at CEGMR for genomic studies. DNA was extracted using QIAAMP genomic DNA according to the manufacturer’s instructions (QIAGEN, USA). A NanoDrop spectrophotometer (Thermo Fisher Scientific, USA) was used to measure the concentration and purity of the DNA for quality assurance. The high-quality DNA was diluted to a concentration of 50–100 ng/μl for Sanger sequencing. Variants were classified according to the American College of Medical Genetics and Genomics (ACMG) guidelines. The reference genome used for the analysis was GRCh38/hg38, and Sanger data interpretation was carried out through finchTV. The families’ histories and pedigrees were used to hypothesise the zygosity and inheritance modes. Finally, segregation analysis was performed on other family members for the same mutations using Sanger sequencing to identify potential carriers.

## Result:

**Fig. 1:**
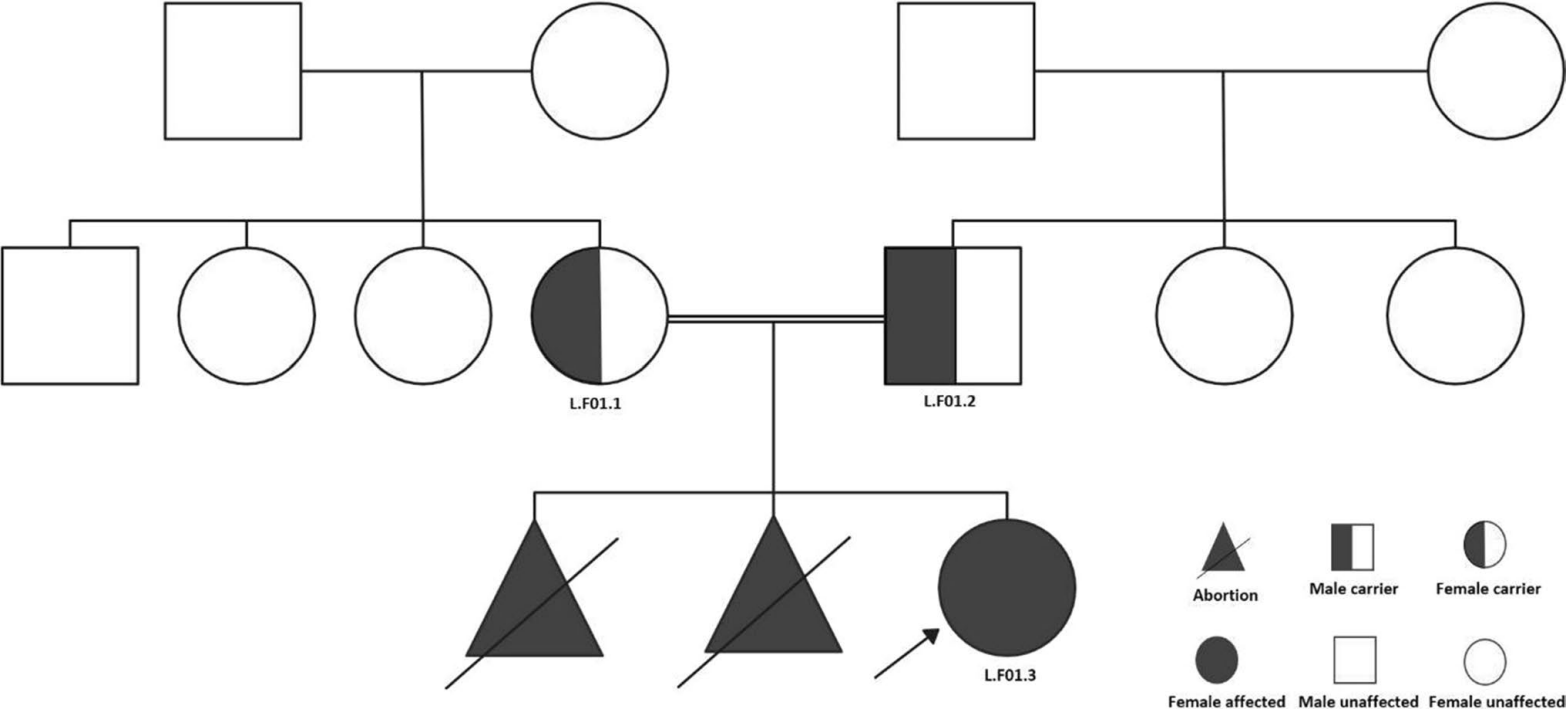
Family one’s pedigree with consanguineous marriage. The proband is indicated by the red colour. L.F01.3 was confirmed to be homozygous for c.1558del in AGPAT2, while the parents (L.F01.1 and L.F01.2) were confirmed to be heterozygous

Six families were recruited from the diabetes clinic. Their phenotypic profiles are found in Supplementary Table 1. Each family’s proband was followed up to check the pattern of inheritance within the family. The first patient was female, as indicated by the number L.F01.3 in Fig. 1. She had an abnormal facial shape, abnormal foot morphology, falangist abnormality of the hand, decreased body weight, a depressed nasal bridge, hirsutism, macrotia, micrognathia, a sacral dimple and a sloping forehead. Genetic testing revealed a mutation in *AGPAT2* c.158del, causing p.(Gly53Alafs*8), NM_006412.4.

**Fig. 2:**
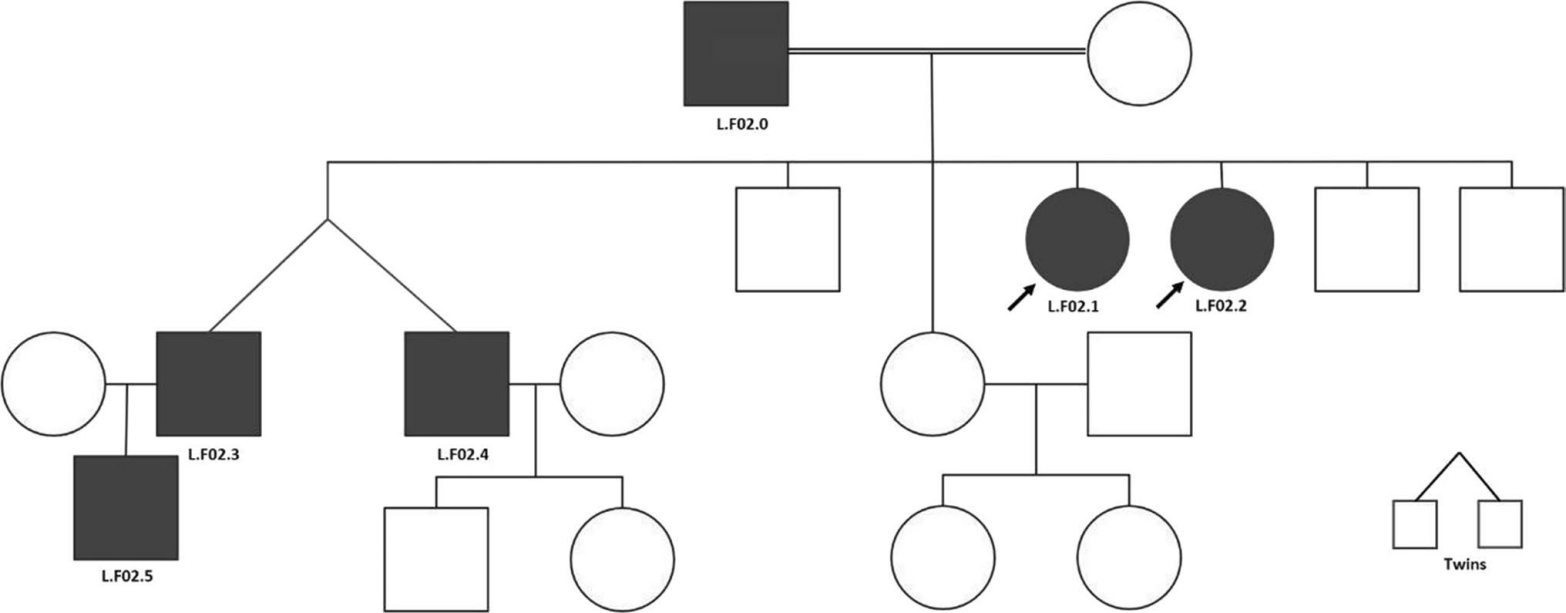
The second family’s pedigree for consanguineous marriage. The black arrow indicates the problem. The two daughters in the red circles were confirmed to be homozygous for c.1558del in AGPAT2. The father, the twin sons and the grandchild were also confirmed to be homozygous for the same mutation

The second patient was also from the same tribe as family 1 and a homozygous carrier for a mutation in *AGPAT2* c.158del causing p.(Gly53Alafs*8), NM_006412.4. The girl, aged 22 years (Fig. 2), had decreased adipose tissue, hepatomegaly, hyperglycaemia, hyperinsulinemia and hypertriglyceridemia. Her parents were first-degree cousins from the mother’s side, and her sister, 16 years old, developed the same phenotype and showed the same mutation. Notably, the father, his twin sons and grandchild showed the same genotype with no phenotype, apart from the father being diagnosed with diabetes.

**Fig. 3:**
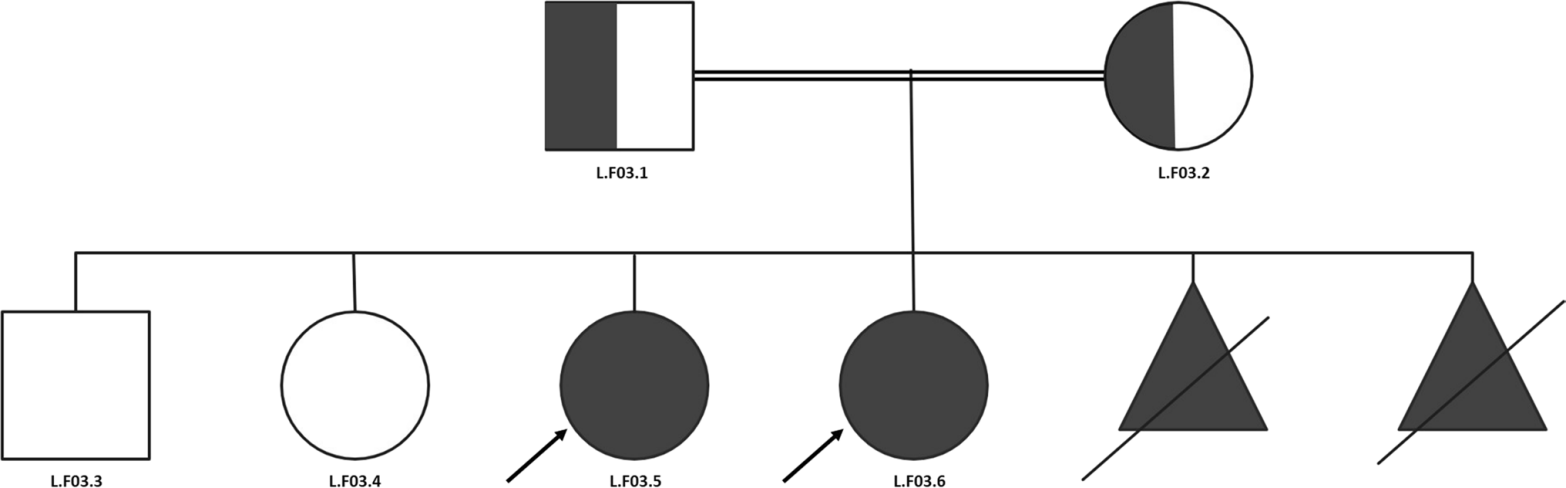
The third family’s pedigree with consanguineous marriage. The proband is indicated by a black arrow. Two daughters (L.F03.5 and L.F03.6) were confirmed to be homozygous for c.942dup in BSCL2, while the parents and a sibling (L.F03.1, L.F03.2 and L.F03.3) were confirmed to be heterozygous, with L.F03.4 being an unaffected daughter

The third patient (L.F03.4, Fig. 3) had pigmentation, delayed speech and language development, Type II diabetes mellitus, elevated hepatic transaminase, hepatic failure, intrauterine growth retardation, renal tubular dysfunction and specific learning disabilities. The genetics test indicated a c.942dup mutation in the BSCL2 gene p.(Leu315Alafs*23) NM_001122955.4. The patient belonged to a consanguineous marriage. Both parents were confirmed heterozygous, and another sister had the same phenotype and genotype.

*.*

**Fig. 4:**
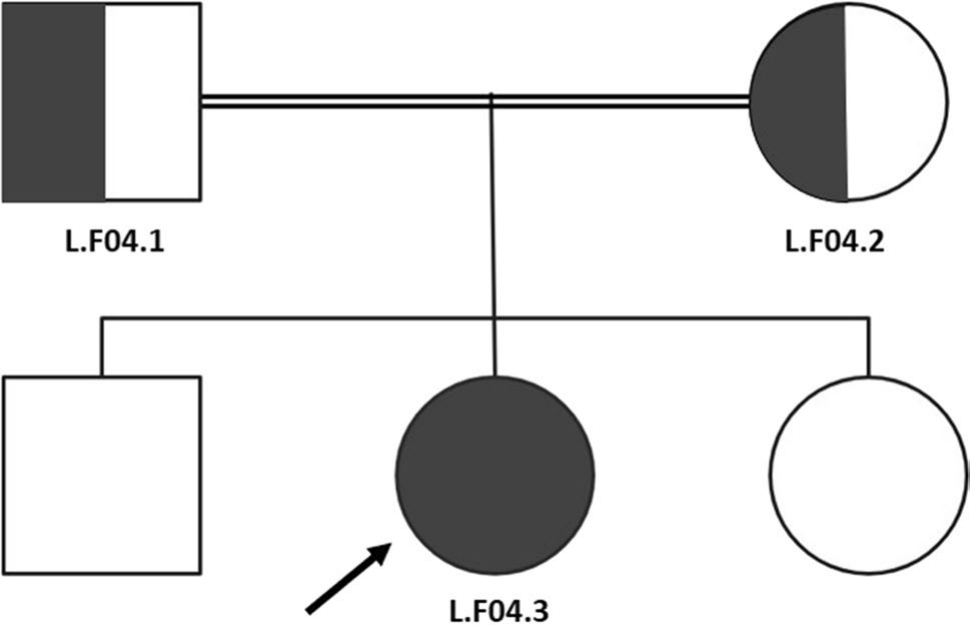
The fourth family’s pedigree with consanguineous marriage. The proband indicated by the black arrow was found to be homozygous for c.158del in the AGPAT2 gene, while both parents were found to be heterozygous

The fourth case was a nine-year-old female from a consanguineous marriage, with *AGPAT2* c.158del, NM_006412.4 causing a change in the protein p.(Gly53Alafs*8) in homozygous form. Both parents were found to be heterozygous, as indicated in Fig. 4.

**Fig. 5:**
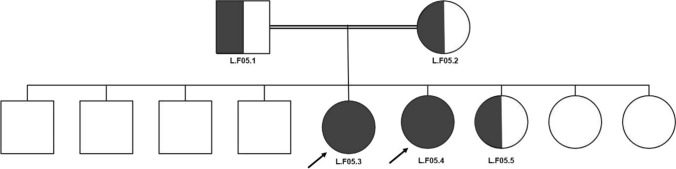
The fifth family’s pedigree with consanguineous marriage. The proband is indicated with a black arrow and red colour. The two daughters in the red circles (L.F05.3 and L.F05.4) were confirmed to be homozygous for c.1558del in AGPAT2, and the parents and one daughter (L.F05.5) were all confirmed to be heterozygous

The fifth family had two children diagnosed and treated with leptin in the diabetes clinic and were confirmed to be homozygous for a mutation in *AGPAT2* c.158del (see Fig. 5), causing a change in the protein p.(Gly53Alafs*8), NM_006412.4.

**Fig. 6:**
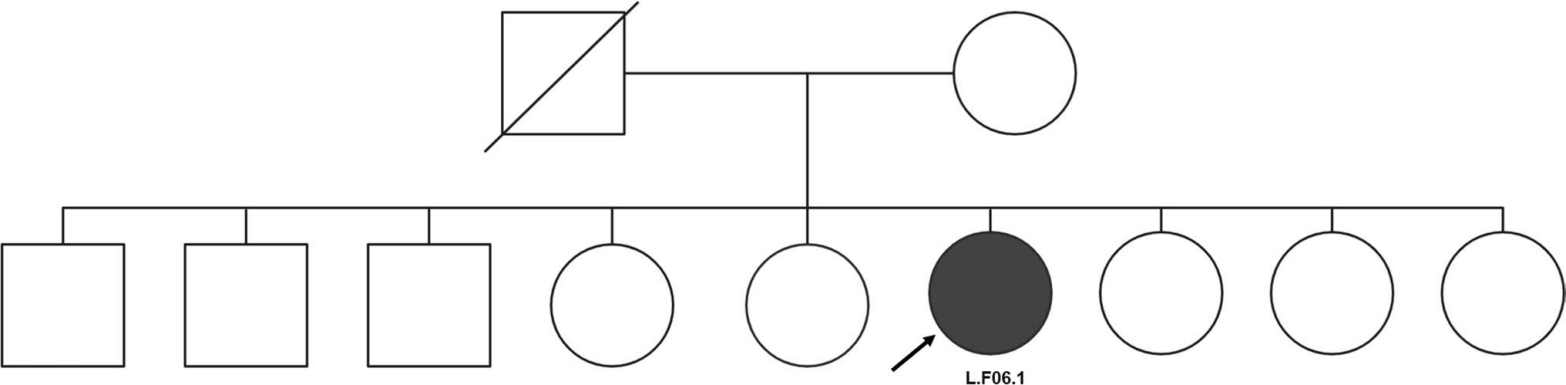
The sixth family’s pedigree with proband L.F06.1 was diagnosed with partial lipodystrophy and indicated with a black arrow. The patient was confirmed to carry a heterozygous mutation for c.1024C > T in PPARG. No other family members were sequenced, and none were reported as having the phenotype

The sixth case was a 32-year-old female with severe hypertriglyceridemia and dyslipidemia as indicated in Fig. 6. She had uncontrolled diabetes mellitus and recurrent pancreatitis. No additional fat was seen in the physical examination. No other family member carried the same phenotype, and the genetics test revealed a heterozygous likely pathogenic c.1024C > T mutation in the *PPARG* gene p.(Gln342Ter), NM_015869.5, which is associated with familial PLD.

## Discussion

There are more than 7362 RDs recognised worldwide [16, 17], with the prevalence found to be one in 2000 healthy individuals. These lifelong conditions require prolonged hospitalisation, rehabilitation and expensive medications that cause financial burdens on healthcare providers and emotional burdens on patients and their families [22]. Notably, 80% of RDs are inherited, and 63% appear in early childhood [23]. In Saudi Arabia, the prevalence of RDs, especially autosomal recessive disorders, is higher due to the high frequency of consanguineous marriages, which increase the chance of offspring inheriting two copies of pathogenic alleles [1, 8].

The Ministry of Health (MoH) in Saudi Arabia has established various programmes, including premarital and newborn screening, to reduce the prevalence of certain genetic diseases [14]. However, these programmes cover only a limited number of the most common conditions [20]. The prevalence of other diseases is still high due to cultural partnering behaviours [4]. Lipodystrophy is an RD with lifelong manifestations.

In this study, we obtained samples from one region, which was limited to patients visiting certain doctors. The other limitation was that not all family members contributed to this study to follow up on the carrier status. Interestingly, all cases were female. However, in Family 2 (Fig. 2), four males carried the same homozygous mutation in the proband with no phenotype representation. This has been observed previously, with clinical findings showing gender differences in lipodystrophy: female patients are more negatively affected than male patients, including earlier onset of severe metabolic abnormalities compared to males (Mcilroy et al. [27]), [24]. This was also observed in familial PLD women who experienced metabolic complications related to insulin resistance more severely than in men (Garg [25]; Hussain and Garg [26]).

Facilitating familial screening for carriers of certain mutations as preventive measures would reduce future incidences and the financial burden of the MoH. In this case, leptin replacement with metreleptin is the golden standard therapy for GLD and PLD [13] at a cost of three million riyals per year for each individual. In this study, we recruited nine individuals diagnosed with GLP and one female with PLD from one region who were treated with Myalept 11.3 MG Cavgene, at a total of 30 million riyals per year, for our study group.

The lack of a national registry for RDs, which can be linked to a global registry to prioritise drug development and research, is urgently needed to monitor the number of cases and disease allele associations [18]. Knowing that particular variations, such as *AGPAT2* c.158del/p.Gly53Alafs*8, are reported only in the Saudi population has led to the proposition of a founder effect [11]. This will accelerate preventive measures in specific regions or focus on certain families to avoid passing the pathogenic allele to future offspring. Implementing a carrier screening for individuals with a family history will prevent increases in the disease rate. If individuals know that they are carriers, this will allow for screening partners before marriage to avoid partnering with another carrier with the same or other mutations in different locations or genes, which can increase the chance of having a child with the phenotype. Furthermore, for Autosomal Domenant carriers, family planning through a genetic counsellor can assess the chances of healthy offspring.

The clinical findings show that there are regular gender differences in lipodystrophy; female patients are more negatively affected than male patients (Mcilroy et al., [27]). Women with familial PLD experience metabolic complications related to insulin resistance more severely than do men. These findings suggest that women who are both regionally and generally obese may also have more severe metabolic aftereffects of insulin resistance (Garg [25]; Hussain and Garg 26).

## Conclusion

This study focused on lipodystrophy, an RD that requires high-cost and prolonged treatment. Given that this disease runs in families, cases increase yearly due to consanguineous marriages. It is alarming that this rare condition could become common within a few years, and it is essential to implement preventive measures to avoid increases in allele frequency in our population. Therefore, a national registry is needed to document the incidence rate in the kingdom, and it is highly recommended that screening programmes for families with RD or ultra-rare diseases be implemented along with educational programmes to promote healthy marriages.

### Supplementary Information

Below is the link to the electronic supplementary material.

Supplementary file1 (XLSX 11 KB)

## Data Availability

Data are available upon request by contacting the PI.
